# Whiplash Patients with Cervicogenic Headache After Lateral Atlanto-Axial Joint Pulsed Radiofrequency Treatment

**DOI:** 10.5812/kowsar.22287523.3590

**Published:** 2012-01-01

**Authors:** Nicholas HL Chua, Willy Halim, Andrea WM Evers, Kris CP Vissers

**Affiliations:** 1 Department of Anesthesiology, Intensive Care and Pain Medicine, Tan Tock Seng Hospital, Singapore; 2 Department of Anesthesiology and Pain Management, St Anna Hospital, Geldrop, The Netherlands; 3 Department of Anesthesiology, Pain and Palliative Medicine, Radboud University, Nijmegen Medical Center, Nijmegen, The Netherlands

**Keywords:** Whiplash Injury, Atlanto-Axial Joint, Pulsed Radiofrequency Treatment, Headache

## Abstract

**Background:**

Whiplash patients regard cervicogenic headache (CEH) as the most burdensome symptom of their condition. Sufferers experience a significant degree of disability from headache, associated neck pain and disability, and sleep disturbance. Lateral C1/2 joint pulsed radiofrequency (PRF) treatment has been shown to produce significant relief from headache in patients with CEH.

**Objectives:**

The objective of this retrospective questionnaire study of 45 consecutive whiplash patients with CEH who had undergone antero-lateral atlantoaxial joint pulsed radiofrequency treatment (AA PRF) was to evaluate the treatment’s long-term effects on pain-related disability and health-related quality of life.

**Patients and Methods:**

Four questionnaires were sent to all 45 patients who had undergone AA PRF: 1) The short form-36 (SF-36); 2) The neck disability index (NDI); 3) The medical outcome scale-sleep scale (MOS-SS); 4) The headache impact test-6 (HIT-6). All 45 patients received AA PRF under fluoroscopic guidance. PRF treatment was conducted at 45 V with a pulsed frequency of 4 Hz and a pulsed width of 10 ms for 4 minutes .

**Results:**

Patients who responded to the procedure reported lower pain scores at 2, 6, and 12 months of follow-up compared to nonresponders. More important, patients reported marked improvements in headache impact (P < 0.01), neck-disability scores (P < 0.01), awakening due to headache (P < 0.01), and sleep problems (9-item; P < 0.05) on the MOS-SS. Responders to the procedure also reported a significantly higher health-related quality of life in terms of bodily pain (P < 0.05) and health change (P < 0.01) on the SF-36.

**Conclusions:**

In light of the inherent limitations of our retrospective study, AA PRF treatment can only be tentatively viewed as a promising treatment modality for whiplash patients with CEH and is subject to validation in future studies.

## 1. Background

The term cervicogenic headache (CEH) was first coined by Sjaastad et al. in 1983. In 1990 the CHISG criteria (cervicogenic headache international study group) for CEH was issued (1). Whiplash injuries were later implicated as likely triggers of CEH (1). Whiplash-associated disorders (WAD) are very costly to society, and patients have rated headaches as the most burdensome WAD (2).

The prevalence of CEH had been estimated as high as 4.1% in the general population and as high as 17.5% among patients with severe headaches. For patients with headaches after whiplash, the prevalence is as high as 53% (3-5).

Most CEH sufferers experience a significant degree of disability from headache, associated neck pain, and sleep disturbance. It is often the disability emanating from CEH attacks that compromises quality of life for these patients. Currently, no drugs are effective for CEH. A randomized controlled study showed that manual therapy alone was no more effective than exercise alone (6, 7). Lateral C1/2 joint injections have identified the lateral C1/2 joint as a source of pain in patients with CEH (8, 9). Narouze et al. (10) found that 25% of their patients experienced 50% pain relief within 3 months. In a retrospective study with 86 patients, pulsed radiofrequency (PRF) application on the antero-lateral C1/2 joint (AA PRF) produced long-term pain relief up to 6 months, with more than 50% of patients experiencing pain relief of more than 50% (11).

Using cervical zygapophysial joint pain as a model for chronic neck pain, Wallis and colleagues showed that all patients who obtained complete pain relief exhibited resolution of their preoperative psychological distress, whereas those who were unrelieved continued to demonstrate signs of psychological distress (12).

## 2. Objectives

This retrospective questionnaire study of 45 WAD patients with CEH who had undergone antero-lateral C1/2 joint PRF application (AA PRF) more than 1 year ago aimed to evaluate its AA PRF’s effects on pain-related disability and health-related quality of life.

## 3. Patients and Methods

Institutional review board approval was obtained prior to administering the questionnaire to all patients. This retrospective questionnaire study included 45 consecutive whiplash patients who had undergone lateral C1/2 joint PRF application for CEH in a single pain center in the Netherlands between January 2007 and February 2009. The patients were recruited from a review of the pain center’s procedure records and verified with the individual patient’s medical records. All 45 patients who had fulfilled clinical criteria specified in Box, had undergone cervical facet denervation (C3 to C5) prior to the antero-lateral C1/2 PRF with minimal improvement. The lateral C1/2 joints in these 45 patients were found to be extremely tender, even after cervical facet denervation. All 45 patients were sent four questionnaires that included the Short Form-36 (SF-36) (13), neck disability index (NDI) (14), the medical outcome scale-sleep scale (MOS-SS) (15), and the headache impact test-6 (HIT-6) (16). All four questionnaires have been established for reliability and validity in the Dutch population (17-20). The patients were also sent a general personal data form that included a dichotomous question of whether they had experienced more than 50% pain relief after receiving the lateral C1/2 joint (AA PRF) injection. After all questionnaires were returned, post-AA PRF progress was evaluated by retrospectively retrieving pain scores (numerical rating scale of 0 to 10) of all 45 patients from individual case files. The NRS scores were retrieved at 2, 6, and 12 months. These data were all collected by an assistant not involved in the design of the study or in the analysis of the data.

**Box.** Clinical Criteria Used in Our Center for the Diagnosis of CE Attributable to Whiplash Injury

Clinical Criteria for Cervicogenic Headache Attributable to Whiplash Injury	
1	Predominantly unilateral headache without side-shift
2	Symptoms and signs of neck involvement: pain triggered by neck movement or external pressure of the posterior neck or occipital region; ipsilateral neck, shoulder, and arm pain; reduced range of motion.
3	Pain episodes of varying duration or fluctuating continuous pain
4	Moderate, non-excruciating pain, usually of a non-throbbing nature
5	Whiplash injury sustained prior to onset of headache with no obvious neurological deficit (Grade II Quebac Task Force classification)
6	No direct head injury or any loss of consciousness

**Figure 1.**
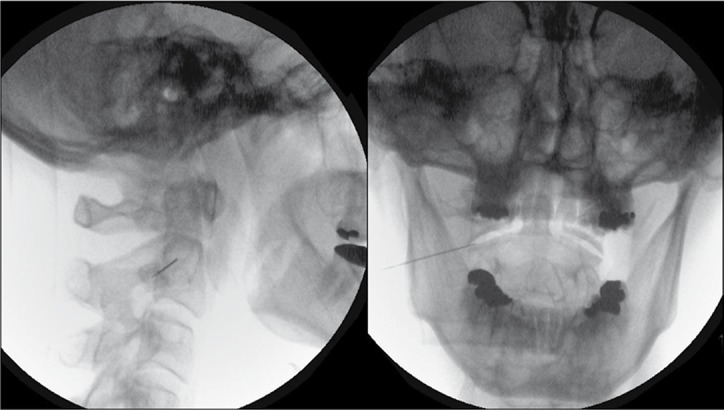
Lateral View With a 5º Oblique Tilt for Initial Needle Entry (Left). Postero-Anterior View (Right) of Needle Entry into Lateral C1/2 Joint. Notice the Active Tip Does Not Contact the Intra-Articular Osseous Surface.

The technical details of the percutaneous procedure have been described elsewhere (11). A 22-G, 45-mm insulated radiofrequency needle with a 5-mm active tip was introduced percutaneously, under fluoroscopic control, so that it entered the lateral 1/3 of the of the antero-lateral C1/2 joint (Figure 1). Guided by fluoroscopy, it is important that the noninsulated needle tip does not contact either intra-articular osseous surface of the lateral C1/2 joint. This is to avoid causing the patient unnecessary pain during sensory stimulation. With the active tip within the intra-articular space, sensory stimulation at 50 Hz up to 1.0 V and motor stimulation at 2 Hz up to 1.0 V is almost always negative. PRF application at 45 V was then initiated with a pulse frequency of 4 Hz, pulse duration of 10 ms for 4 minutes . Wte do not routinely give contrast, local anesthetic, or steroids either before or after the PRF application.

For the analysis of the MOS sleep scale, 90% completion of a section was considered sufficient for analysis. The HIT-6 and NDI scores were excluded if one item was missing. For the SF-36 subscale scores, missing values were substituted with group mean values in accordance with the instructions in the SF-36 manual. All statistics were performed using SPSS (version 16.0 for Windows). Descriptive statistics were generally reported as mean values ± 1 standard deviation (SD) and were analyzed for their degree of skewness or kurtosis. A student’s t-test (continuous variables) and Chi-square test (for dichotomous variables) were used to compare the differences in baseline characteristics and study measures between both groups. Pearson’s correlation coefficients were used to evaluate the correlation between questionnaire scores and their relevant domains. A significance level of P < 0.05 was used for all tests.

## 4. Results

Thirty-six patients returned their questionnaires within 4 weeks. We attempted to contact the remaining 9 patients. Four patients returned their questionnaires after 2 reminders (88.9%). We were unable to contact 1 patient. One patient agreed to the HIT-6 over the phone but not the rest of the questionnaires. Three patients agreed to the use of retrospective data but not to the questionnaires. Forty patients completed the SF-36 and NDI questionnaires, 39 patients completed the MOS-SS questionnaires, while 41 patients completed the HIT-6 questionnaires. Of the 44 patients who consented to the study, 25 patients self-reported more than 50% pain relief at the time of the survey and were denoted as treatment responders (hereafter, responders). The remaining 19 patients reported less than 50% pain relief at the time of the survey and were denoted as treatment nonresponders (hereafter, nonresponders). The responders’ post-AA PRF improvement in pain scores was consistently lower than the scores of the nonresponders at 2, 6, and 12 months (Table 1; P < 0.05). The baseline demographic characteristics of the responders and nonresponders did not differ significantly (Table 2). Additionally, the history of postprocedure employment, litigation, and government benefits did not differ either; the only demographic characteristic that did vary significantly was the age of the responders (t = -1.95, P < 0.058).

**Table 1.** Questionnaire and Pain (NRS) a Scores

	Responder Group, (n = 25)	Non-Responder Group, (n = 19)	t score	*P* value
HIT-6 ^a^, Mean ± SD	56.7 ± 11.7	68.6 ± 7.1	4.02	< 0.001 ^b^
NDI ^a^, Mean ± SD	18.9 ± 8.4	27.1 ± 7.4	3.27	0.002 ^b^
MOS-SS ^a^, Mean ± SD				
Sleep disturbance	44.8 ± 24.9	55.3 ± 25.3	1.30	0.203
Snoring	42.9 ± 28.5	29.4 ± 33.3	-1.32	0.196
Headache	44.5 ± 30.2	72.9 ± 28.2	3.02	0.005 ^b^
Sleep adequacy	43.2 ± 28.7	32.4 ± 27.0	-1.21	0.235
Somnolence	35.8 ± 25.9	46.7 ± 20.7	1.46	0.152
Sleep problems index I	41.2 ± 10.4	48.2 ± 9.1	2.25	0.030 ^b^
Sleep problems index II	42.5 ± 13.2	51.1 ± 10.7	2.24	0.031 ^b^
SF-36, Mean ± SD				
Physical functioning	63.9 ± 23.9	57.4 ± 18.2	-0.98	0.331
Role-physical	37.0 ± 43.2	16.2 ± 26.4	-1.88	0.068
Bodily pain	55.8 ± 23.0	41.1 ± 15.3	-2.44	0.020 ^b^
General health	53.3 ± 23.3	49.7 ± 21.8	-0.50	0.623
Vitality, Mean ± SD	46.5 ± 21.8	42.4 ± 17.0	-0.68	0.500
Social functioning, Mean ± SD	61.4 ± 25.3	57.4 ± 16.0	-0.62	0.538
Role-emotional, Mean ± SD	76.8 ± 38.2	58.3 ± 41.3	-1.42	0.166
Mental health, Mean ± SD	70.3 ± 20.6	64.7 ± 18.6	-0.89	0.379
Perceived health change, Mean ± SD	65.2 ± 26.9	39.7 ± 17.8	-3.60	0.001 ^b^
NRS a scores, Mean ± SD				
0 month	8.68 ± 0.78	8.32 ± 0.82	1.46	0.153
2 months	1.64 ± 1.53	6.00 ± 2.85	-5.98	< 0.001 ^b^
6 months	1.68 ± 1.89	6.53 ± 2.41	-7.08	< 0.001 ^b^
12 months	1.45 ± 1.41	7.74 ± 1.32	-14.71	< 0.001 ^b^

^a^ Abbreviations: HIT-6; headache impact test-6; MOS-SS, medical outcome scale-sleep scale; NDI, neck disability index; NRS, numerical rating scale

^b^ Denotes comparisons that are statistically significant at P < 0.05.

**Table 2.** Baseline Characteristics of Treatment Responders and Nonresponders to Antero-Lateral C1/2 Joint PRF^a^

	Non-Responders, (n = 19)	Responders, (n = 25)	P value
Age, y, Mean ± SD	41 ± 13	49 ± 11	0.05
Gender, No.			0.68
Male	11	16	
Female	8	9	
Height, cm, Mean ± SD	174 ± 9	171 ± 7	0.25
Weight, kg, Mean ± SD	73.1 ± 14.5	70.0 ± 15.3	0.81
Secondary education and above, No.	3	7	0.32
Smoke, No.	7	4	0.12
Alcohol, No.	7	10	0.79
Years of pain, Mean ± SD	6.9 ± 9.4	7.1 ± 3.5	0.66
Pre-procedure numeric rating scale (NRS)- score, Mean ± SD	8.4 ± 0.8	8.6 ± 0.8	0.38
Involved in litigation prior to procedure, No.	2	4	0.59
Years post-procedure, Mean ± SD	2.0 ± 0.5	1.7 ± 0.7	0.39
Currently employed, No.	9	10	0.59
Returned to work, No.	10	11	0.82
Benefits act from work loss or injury, No.	7	7	0.52

^a^ Abbreviation: PRF, pulsed radiofrequency

The mean questionnaire scores (± SD) of both the responder and the nonresponder group are shown in Table 1. The HIT-6 and the NDI scores were significantly lower in the responder group than in the nonresponder group. The domains of awakening due to headache sleep problems Index I (6-items) and sleep problems Index II (9-items) in the MOS-SS were all significantly lower in the responder group than in the nonresponder group. Responders also had higher mean scores in all domains of the SF-36 (Table 1). However, this achieved statistical significance in only 2 subscales: bodily pain (t = -2.44, P < 0.05) and perception of health change (t = -3.60, P < 0.01), with role-physical being nonsignificant (t = -1.88, P = 0.68).

The lower headache impact scores in the responder group correlated significantly with a decrease in neck disability (r = 0.64, P < 0.001) as well as with awakening due to headache (r = 0.55, P < 0.01) in the MOS-SS. The lower neck-disability score in the responder group also correlated significantly with a decrease in sleep problems and awakening due to headache (6-item: r = 0.36, P < 0.05; 9-item: r = 0.44, P < 0.01) in the MOS-SS. The perceived improvement in health correlated well with a reduction in the impact of headaches on life (r = -0.54, P = 0.001), neck disability (r = -0.50, P = 0.001), and bodily pain (r = 0.67, P < 0.001).

## 5. Discussion

From our retrospective findings, patients with sustained pain relief after AA PRF experienced improvements in headaches’ impact on life, reductions in neck disability, improvements with respect to sleep problems, and an improved overall perception of health within 12 months after treatment. The improvements in headaches’ impact on life were also highly correlated with improvements in neck disability and sleep. The divergent pain scores between the responders and nonresponders at 2, 6, and 12 months after AA PRF were reinforced by a self-reported improvement in general health by the responders. Despite consistently higher scores in all the health-related, quality-of-life domains, we were limited by a relatively small sample size to detect significant improvements in those who responded to AA PRF. We are unable to conclude that the extended duration of pain relief observed in the responder group is entirely a result of antero-lateral C1/2 joint PRF due to inherent limitations in our retrospective study. However, our findings suggest that if whiplash patients with CEH do respond to intra-articular lateral C1/2 joint PRF, they may not only improve in terms of pain scores but also may exhibit positive changes to life burdens, neck-related disability, and perceived health over the long term.

An extensive body of research is looking at the mechanisms through which PRF acts. At the time of this writing, most studies point towards an alteration in synaptic transmission in a neuromodulatory-type effect (20-22). The effects of PRF were initially postulated to be via a combination of excitatory C-fibre response suppression as well as inhibition of synaptic transmission with the decrease in excitatory postsynaptic potential (22-24). However, in intra-articular PRF, this is unlikely to be the case: the effects of intra-articular PRF are most likely a result of its anti-inflammatory properties. This occurs as a result of the attenuation of proinflammatory cytokines such as interleukin (IL)-1b, tumor necrosis factor a (TNF-a), and IL-6 by the generated electric fields (25, 26). In fact, IL-1b, which is present in high amounts in OA cartilage, is considered to be one of the main catabolic factors involved in the cartilage matrix degradation (27, 28). In addition, an up-regulation of adenosine A2a receptor density has been observed in human neutrophils treated with pulsed electric fields (29). Activation of adenosine A2a receptors seems to be associated with inhibition of the catabolic cytokines TNF-a, IL-6, and IL-8 (30, 31). It seems intuitive to presume a similar mechanism of action of the A2a receptor on chondrocyte membranes, with a similar consequential effect of cytokine inhibition (27, 31).

One of the hypotheses generated from this retrospective study is thus the chrondro-protective mode of action of intra-articular PRF, which may explain the anecdotal observation of pain relief 2 to 4 weeks after PRF in a number of patients. A number of in-vitro studies have shown that chondrocyte proliferation and matrix synthesis are significantly enhanced by pulsed electrical fields (28, 32-34). Fini et al. (27) suggest that the delivery of pulsed electromagnetic fields combines an anabolic effect on chondrocytes, a catabolic cytokine blockage, a stimulatory effect on anabolic cytokine production, and a counteraction of the inflammatory process in osteoarthritis. Cosman et al. (35) assert that magnetic fields generated in PRF are negligible and any therapeutic effects are due to the electric fields. More research will therefore be required to verify in-vitro effects, if this hypothesized chondro-protective mechanism is indeed true.

The main limitation of our study is the lack of a control group. The retrospective nature of the study and the relatively small sample size also prevent strong conclusions regarding the efficacy of antero-lateral C1/2 PRF for whiplash patients with CEH.

As we attempt to prospectively evaluate our results in a formal trial, more studies will be also needed to evaluate other treatment modalities in this multifaceted clinical diagnosis. It seems prudent to adopt an algorithmic approach in the management of such patients, and at the time of this writing, antero-lateral C1/2 joint PRF should be at most be regarded as a potentially viable treatment modality subject to validation in future studies.
